# Assessment of Border Control Measures and Community Containment Measures Used in Japan during the Early Stages of Pandemic (H1N1) 2009

**DOI:** 10.1371/journal.pone.0031289

**Published:** 2012-02-15

**Authors:** Hiroko Sakaguchi, Masashi Tsunoda, Koji Wada, Hiroshi Ohta, Masatoshi Kawashima, Yae Yoshino, Yoshiharu Aizawa

**Affiliations:** 1 Department of Occupational Health, Graduate School of Medical Sciences, Kitasato University, Sagamihara, Kanagawa, Japan; 2 Department of Preventive Medicine, Kitasato University School of Medicine, Sagamihara, Kanagawa, Japan; 3 Department of Public Health, Kitasato University School of Medicine, Sagamihara, Kanagawa, Japan; University of Hong Kong, Hong Kong

## Abstract

**Background:**

In the early stages of Pandemic (H1N1) 2009, border control measures were taken by quarantine stations to block the entry of infected individuals into Japan and community containment measures were implemented to prevent the spreading. The objectives of this study were to describe these measures and the characteristics of infected individuals, and to assess the measures' effectiveness.

**Methodology/Principal Findings:**

Border control and community containment measures implemented from April to June (Period I: April 28–May 21, Period II: May 22–June 18) 2009 were described. Number of individuals identified and disease characteristics were analyzed. For entry screening, a health declaration form and an infrared thermoscanner were used to detect symptomatic passengers. Passengers indicated for the rapid influenza test underwent the test followed by RT-PCR. Patients positive for H1N1 were isolated, and close contacts were quarantined. Entry cards were handed out to all asymptomatic passengers informing them about how to contact a health center in case they developed symptoms. Nine individuals were identified by entry screening and 1 during quarantine to have Pandemic (H1N1) 2009. Health monitoring by health centers was performed in period I for passengers arriving from affected countries and in period II for those who had come into contact with the individuals identified by entry screening. Health monitoring identified 3 infected individuals among 129,546 in Period I and 5 among 746 in Period II. Enhanced surveillance, which included mandatory reporting of details of the infected individuals, identified 812 individuals, 141 (18%) of whom had a history of international travel. Twenty-four of these 141 passengers picked up by enhanced surveillance had been developing symptoms on entry and were missed at screening.

**Conclusion/Significance:**

Symptomatic passengers were detected by the various entry screening measures put in place. Enhanced surveillance provided data for the improvement of public health measures in future pandemics.

## Introduction

Once a novel influenza strain emerges in a country, international travel from that country can present a risk for infection in other countries [1], [2]. As the pathogenicity and infectivity of new strain are unclear in the early stages of emergence, proactive public health measures are required to block the entry of infected individuals from abroad and delay the spread of infection in communities [3], [4]. In the case of Pandemic (H1N1) 2009 an influenza strain that emerged in Mexico and the U.S. in 2009 [5]–[8], many countries implemented border control measures for travelers from affected countries upon their arrival [9], . These border control measures have been effective, especially for island countries, because it was practicable to block the entry at critical points–at airports and harbors–and, once in the country, to keep track of the whereabouts of individual passengers who entered from affected countries [11], [12].

Japan was among the countries that implemented border control measures for international travelers, in accordance with the “Guidelines for the Prevention and Control of Pandemic Influenza” [13] proposed by a governmental committee in February 2009. After the World Health Organization (WHO) declared a pandemic alert phase 4, all quarantine stations in Japan, in accordance with the Guidelines and the Quarantine Act, performed entry screening as part of border control measures. As Cowling et al. pointed out in their review of entry screening policies adopted by different nations during the pandemic, individuals were identified by various methods according to the measures each nation put in place [10]. Although specific measures were adopted, including entry screening in airplane cabins and quarantine of individuals who had come into close contact with infected individuals, details of their assessment remain unclear.

As the incubation period for the influenza virus is reported to be 2 days on average (range: 1–4 days) [14], [15], it is difficult to identify infected individuals during the incubation period through border control measures alone. Blocking the entry of all infected individuals from other countries is almost impossible to achieve and therefore it is necessary to conduct health monitoring, which traces individuals who may possibly develop symptoms after entry into the country. Another important measure to prevent the infection spreading in communities is enhanced surveillance, which includes proactive examinations to identify patients and mandatory reporting of patient information [16].

To establish optimal measures for the future, it is necessary to clarify what border control measures and community containment measures were implemented in Japan during the early stages in the outbreak of Pandemic (H1N1) 2009 and how effective they were. At present, there is only a brief description in Japanese of such measures [17], and an assessment of the measures has not yet been made.

Therefore, in this study, we analyzed in detail the border control and community containment measures that were put into effect and the characteristics of individuals identified by the respective measures during the early stages in the outbreak of Pandemic (H1N1) 2009. Especially important to clarify were the following: (1) the methods used to identify individuals through the respective measures implemented, the numbers and characteristics of the individuals screened, and the standards for the indication for the influenza diagnosis test; (2) the information that was obtained, at critical points of entry, for arriving passengers; and (3) the definition of “close contact with infected individuals” in relation to individuals for whom this was deemed to apply. In addition, we reviewed those cases where individuals already infected at entry were missed by the screening process put in place. We undertook the above with the aims of assessing these measures and suggesting how to better manage pandemics in the future.

## Results

**Figure 1:**
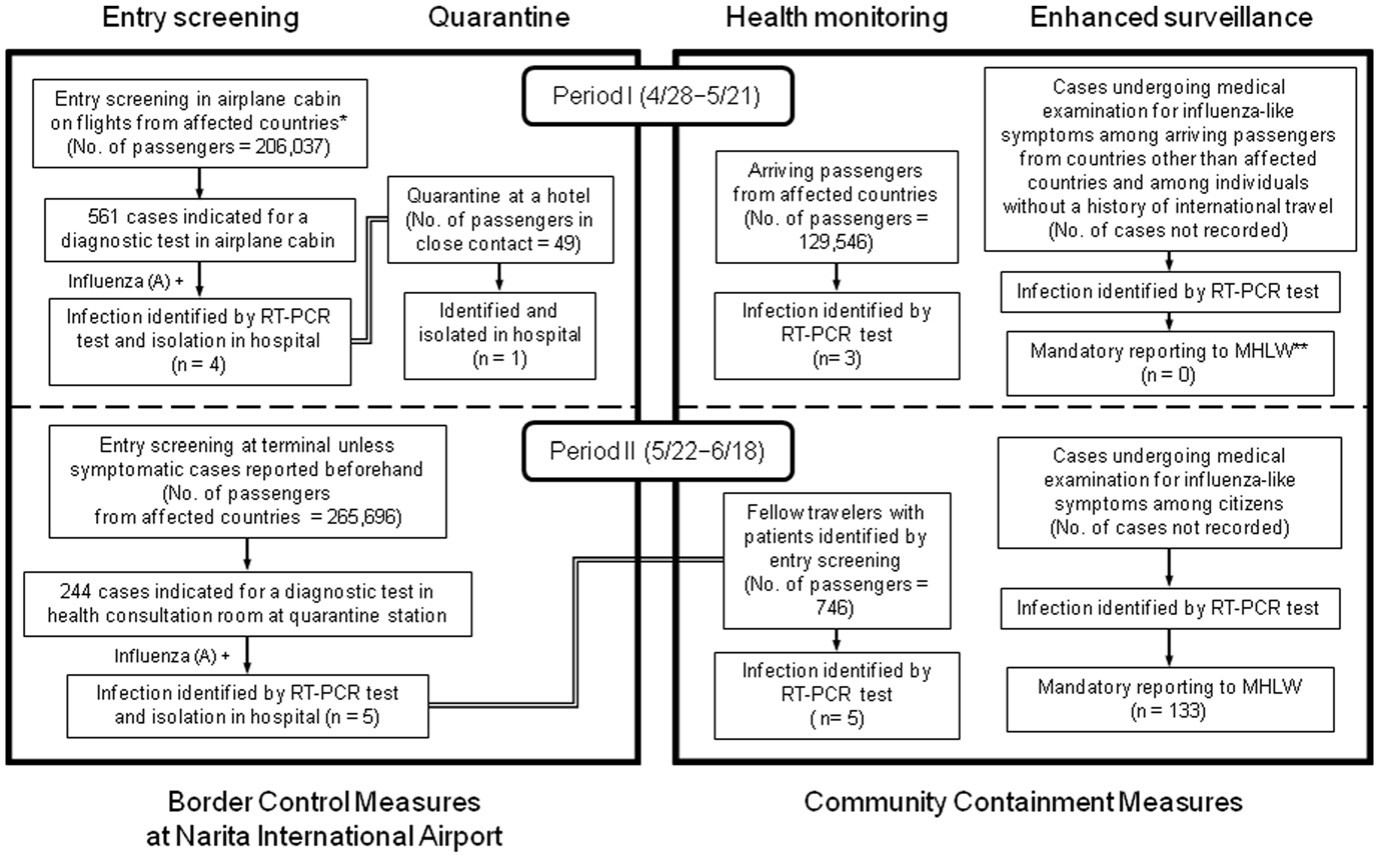
Flow of each measure to identify infected individuals in Japan during the period April 28–June 18 and the number of individuals infected with Pandemic (H1N1) 2009 with international travel histories within 7 days of onset (n = 151). Note: n is the number of individuals identified with Pandemic (H1N1) 2009 by each measure: 10 for Border Control Measures at Narita International Airport (4 from entry screening and 1 from quarantine in period I, 5 from entry screening in period II); 141 for Community Containment Measures (3 from health monitoring in period I, 5 from health monitoring and 133 from mandatory reporting in period II). The 24 missed cases are not shown as it is not known whether they were identified by health monitoring or enhanced surveillance. *Affected countries: Mexico, mainland U.S., Canada. **MHLW: Ministry of Health, Labour and Welfare of Japan.

The flow for identifying patients by border control measures and community containment measures during Periods I and II are shown in Figure 1. Details of these measures are described below. The numbers of infected individuals identified by each measure are given in Figure 1.

### Details of border control measures

#### Entry screening

During entry screening, passengers arriving from other countries had their surface body temperature taken with an infrared thermoscanner (e.g., TVS-500EX, NEC Avio Infrared Technologies Co., Ltd., Tokyo, Japan [18]) by quarantine officers in both Periods I and II. In airplane cabins, a handheld infrared thermoscanner was pointed at stationary passengers and crew members. Inside the airport terminal building, a fixed infrared thermoscanner was pointed at passengers walking past. Instead of asking passengers to remove any glasses they were wearing which could potentially influence the results, the infrared thermoscanner was targeted carefully on their face by quarantine officers. Mexico, the mainland U.S., and Canada were classified as “affected countries”. Quarantine officers collected the self-reporting health declaration forms that had been handed out in the airplane cabin to all the passengers arriving on planes from these countries and in the airport terminal to passengers arriving on planes from other countries. The form contained items on the presence or absence of any symptoms, history of contact with infected individuals, and the places they planned to visit in Japan during the first 10 days after entry. An entry card was then given to all arriving passengers instructing them to consult with staff at public health centers if they developed symptoms while in Japan. The card also certified completion of entry screening. In Period I, Japanese quarantine officers boarded direct flights arriving from the affected countries. In Period II, when the quarantine station received information from the airlines that symptomatic individual was onboard a flight, entry screening was performed in the airplane cabin before disembarkation. Moreover, if any individuals had declared influenza-like symptoms on the health declaration forms collected, doctors would examine the individuals to assess whether they were indicated for the rapid influenza diagnostic test, which could distinguish influenza types A and B: this test was carried out in the airplane cabin in Period I and in a health consultation room at the airport's quarantine station in Period II. The standards for the indication for the rapid influenza diagnostic test have been established as follows: passengers with travel history to the affected countries who have 2 or more of 4 symptoms (1. nasal discharge or nasal obstruction, 2. sore throat, 3. cough, and 4. fever, or feeling feverish and chills) or passengers with body temperature >38°C (directly measured by tympanic or axillary temperature) [13]. At entry screening, all individuals who underwent the rapid influenza diagnostic test were reported to the Ministry of Health, Labour and Welfare (MHLW). Those individuals who did not meet the standards for the indication for testing were not reported to MHLW and also did not undergo testing during entry screening. If the results of the rapid influenza diagnostic test were positive for type A, individuals suspected of having Pandemic (H1N1) 2009 were transferred to designated medical institutions for isolation. If they were identified as having Pandemic (H1N1) 2009 by the RT-PCR test, they were treated and isolated at these institutions until no excretion of the virus was confirmed. In addition to the 200 staff or so at Narita Airport Quarantine Station who were involved in implementing these measures, staff from other airports, ports, the Self-Defense Force, and national hospitals were also involved.

#### Quarantine

Quarantine was a measure taken in Period I for travelers who had been in close contact with a symptomatic individual identified on entry screening to be infected with Pandemic (H1N1) 2009. According to the Guidelines for the Prevention and Control of Pandemic Influenza [13], close contact was defined as a person (passenger) seated within a radius of 2 m (up to 3 seats away in all directions) from a symptomatic individual with suspect infection and who had traveled the same course as the symptomatic individual. If a symptomatic individual was identified by the RT-PCR test to be infected with Pandemic (H1N1) 2009, those who had come into close contact with him or her were quarantined at designated hotels near the airport. The asymptomatic individuals were placed under medical observation in separate rooms at the hotel. Prophylactic medication with an anti-influenza virus was offered during the quarantine period. The quarantine period was set as that of the incubation period of Pandemic (H1N1) 2009: from April 28, the period of quarantine was a maximum of 10 days, which was shortened to a maximum of 7 days from May 15 onwards. When an individual developed influenza-like symptoms during quarantine and the infection was confirmed by the RT-PCR test, the individual was isolated in a designated medical institution.

### Infected individuals identified by border control measures

Table 1 summarizes the data on the number of individuals who underwent entry screening, individuals who were indicated for the rapid influenza diagnostic test at entry screening, positive cases, and individuals identified by the RT-PCR test in Periods I and II from among all passengers arriving on direct flights from affected counties. In Period I, 6 individuals were found to be influenza type-A positive; 4 were confirmed as being infected with Pandemic (H1N1) 2009 by the RT-PCR test and the remaining 2 were determined to have seasonal influenza (H1 and H3, respectively). In Period II, 10 individuals were found to be influenza type-A positive; 5 were confirmed to have Pandemic (H1N1) 2009, 3 had H3, and the remaining 2 had seasonal influenza.

**Table 1:** Among passengers arriving on direct flights from affected countries, number of individuals who underwent entry screening*, number indicated for the rapid influenza diagnostic test, number of positive cases, and number of individuals identified with Pandemic (H1N1) 2009 by RT-PCR tests at Narita Airport Quarantine Station.

	Individuals undergoing entry screening among arriving passengers on direct flights from affected countries (n)	Individuals indicated for rapid influenza diagnostic test** (n)	Individuals found to be influenza type-A positive by rapid influenza diagnostic tests (n)	Individuals identified with Pandemic (H1N1) 2009 by RT-PCR test (n)	Individuals identified with Pandemic (H1N1) 2009 among those indicated for rapid influenza diagnostic test (%)
Period I (in the cabin)					
28 April–7 May	81,941	287	2	0	0.0
8–14 May	63,188	171	3	3	1.8
15–21 May	60,908	103	1	1	1.0
Total	206,037	561	6	4	0.7
Period II (at the quarantine station in the airport terminal)					
22–28 May	61,525	95	4	3	3.2
29 May–4 June	63,757	65	1	0	0.0
5–11 June	66,871	44	4	2	4.5
12–18 June	73,543	40	1	0	0.0
Total	265,696	244	10	5	2.0

*Including transit passengers.

**Passengers with travel history to the affected countries who have ≥2 of 4 symptoms (1. nasal discharge or nasal obstruction, 2. sore throat, 3. cough, and 4. fever, or feeling feverish and chills) or have hyperthermia >38°C (directly measured by the tympanic or axillary temperature).

Table 2 summarizes the characteristics of 10 cases identified as having Pandemic (H1N1) 2009 during entry screening at Narita International Airport, including one individual who was a fellow traveler with the first 3 infected individuals confirmed in Japan and who developed symptoms during the quarantine on the day after arrival. These 3 cases detected for the first time in Japan were from the same party of travelers (1 teacher and 2 students on a school trip) arriving on May 8 from Canada. They had all already developed symptoms 2 days previously. On May 25, an American father and his 5-year-old son, both of whom were residents of Japan, returned from the mainland U.S. The son had developed symptoms 4 days before arriving in Japan and had been diagnosed with Pandemic (H1N1) 2009 before departure. The father also developed symptoms on the day before arrival. Two of the 9 cases detected at entry screening had developed fever >38°C. During Period I, 49 individuals (including the 1 individual who developed Pandemic (H1N1) 2009 mentioned above) were quarantined because of having come into close contact with the first 3 infected individuals detected on May 8. Of those 49 individuals, 36 were fellow travelers in the same school-trip party. The seats on the airplane of 13 of these individuals were within 3 seats away from each other in all directions.

**Table 2:** Characteristics of individuals identified with Pandemic (H1N1) 2009 by border control measures.

Case	Arrival date	Onset date	Sex	Age group	Nationality	Affected country visited	Fellow travelers	Body temperature upon entry screening (°C)
1	8 May	6 May	M	40–49 y	Japanese	Canada	Cases 1–4: traveling together	38.5
2	8 May	6 May	M	10–19 y	Japanese	Canada		36.6
3	8 May	5 May	M	10–19 y	Japanese	Canada		37.1
4	8 May	9 May	M	10–19 y	Japanese	Canada	During quarantine	Afebrile
5	21 May	18 May	M	20–29 y	Korean	Mainland U.S.	None	38.4
6	24 May	23 May	M	40–49 y	Japanese	Mainland U.S.	None	37.1
7	25 May	24 May	M	30–39 y	American	Mainland U.S.	Cases 7 and 8: family traveling together	37.2
8	25 May	21 May	M	5 yrs	American	Mainland U.S.		36.6
9	9 June	8 June	F	10–19 y	Japanese	Canada	Cases 9 and 10: traveling together	36.9
10	9 June	8 June	F	10–19 y	Japanese	Canada		36.8

### Details of community containment measures

#### Health monitoring

Health monitoring by public health centers was performed for all passengers arriving from the affected countries during Period I. In Period II, health monitoring was performed mainly for fellow travelers of infected individuals identified during entry screening. Information on individual passengers based on the health declaration forms collected by quarantine officers was provided by the Quarantine Station to 510 public health centers as of April 2009 for passengers that would stay in their area. Health monitoring was performed for 10 days from April 28 to May 14 and for 7 days from May 15 to June 18. Staff at the public health centers monitored the health of identified individuals by telephone and gave instructions for body temperature to be measured twice a day, in the morning and evening. When any symptoms developed, the individual was asked to report immediately to a public health center. If influenza-like symptoms were confirmed by health monitoring, the public health center arranged a consultation with a doctor in a fever clinic. Fever clinics were special clinics established by prefectural and city governments where patients with pandemic influenza and those they have been in contact with could be assessed, in order to minimize influenza transmission in the community and in other healthcare facilities. At these clinics, infection with Pandemic (H1N1) 2009 was confirmed by the RT-PCR test and individuals were then isolated at a medical institution until no excretion of the virus was confirmed.

#### Enhanced surveillance

Enhanced surveillance was performed in the community. When patients were identified by the RT-PCR test during Periods I and II, medical institutions mandatorily reported these to the national authorities via public health centers. On May 11, 2009, through a TV commercial and the internet, the Prime Minister asked for the people's cooperation, that if an individual living in Japan developed influenza-like symptoms, he or she would consult a public health center. When public health center staff suspected infection, they contacted a doctor to arrange an examination at a fever clinic. In the fever clinic, the rapid influenza diagnostic test was performed, and when type A was detected, it was followed by the RT-PCR test. The following items were to be reported mandatorily: date of onset, symptoms, place of infection, personal characteristics, and date of confirmation of infection with Pandemic (H1N1) 2009 by the RT-PCR test. If an individual had a history of international travel within several days before onset, optional information such as the date of arrival and symptoms at entry were also reported. In the communities where individuals were detected, preventive measures against the spreading of infection were implemented. These included school closures, cancellation of group activities, and administration of prophylactic medication with an anti-influenza virus agent.

### Infected individuals identified by community containment measures

During the 24-days period when health monitoring was being performed for arriving passengers from affected countries in Period I, 117,553 individuals were reported by the Narita Airport Quarantine Station to the public health centers. The total number of individuals including those from other airports across the country was 129,546 according to data compiled by MHLW. Among them, 3 individuals developed symptoms during health monitoring and were confirmed to have Pandemic (H1N1) 2009 by the RT-PCR test. For the individuals indicated for health monitoring during Period II (mainly fellow travelers), 5 of 746 individuals were confirmed to be infected across the whole country. It was reported that 19 individuals did not make contact with a public health center in Period II; the number in Period I is unknown.

There were 812 individuals who developed symptoms between April 28 and June 18 and whose infection with Pandemic (H1N1) 2009 was confirmed by the RT-PCR test in accordance with the community containment measures. During this period, those who were instructed to consult a fever clinic and those who underwent rapid influenza diagnostics tests under these community containment measures are unknown. Among those 812 individuals, 141 (18.4%) had a history of international travel within 7 days before symptom onset. The demographic profiles of these 141 patients are shown in Table 3. Among these, 68 (48.2%) had visited the mainland U.S., 37 (26.2%) had visited Hawaii, and 23 (16.3%) had visited the Philippines.

**Table 3:** Demographic profiles of individuals identified with Pandemic (H1N1) 2009 with travel histories confirmed by community containment measures (n = 141).

	Patients (n)	%
Sex		
Male	65	46.1
Female	76	53.9
Age group (years)		
0–9	29	20.6
10–19	24	17.0
20–29	38	27.0
30–39	28	19.9
40–49	9	6.4
50–59	9	6.4
≥60	4	2.8
Country visited within 7 days of developing symptoms		
Mainland U.S.	68	48.2
Hawaii	37	26.2
Philippines	23	16.3
Canada	5	3.5
Australia	4	2.8
Hong Kong	1	0.7
China	1	0.7
Brazil	1	0.7
Thailand	1	0.7
Days from arrival to onset*		
−4	2	1.4
−3	3	2.1
−2	2	1.4
−1	4	2.8
0	32 (13**)	22.7
1	36	25.5
2	24	17.0
3	25	17.7
4	8	5.7
5	4	2.8
6	1	0.7

*Days from arrival to onset is expressed as 0 for the day of arrival, as minus number of days for previous days, and as plus number of days for following days.

**Number of symptomatic individuals upon entry.

Twenty-four of the 141 individuals had symptoms upon arrival. Table 4 shows the characteristics of these 24 individuals reported in community containment measures, who were not identified at entry screening despite having at least one of the four named symptoms on arrival. There were 16 and 8 individuals with onset on the day of arrival and on previous days, respectively. The results of the rapid influenza diagnostic test during entry screening showed 5 individuals were negative for infection. Twelve individuals had fever >38°C at entry screening, 2 of whom had visited the Philippines and were suspected of having Dengue fever. They were tested for Dengue fever but not for Pandemic (H1N1) 2009. Although 3 individuals stated they were asymptomatic on their health declaration forms (Cases 6, 13, and 15), case 13 was detected by the infrared thermoscanner to have high fever.

**Table 4:** Characteristics of individuals with developing symptoms upon entry and identified as having Pandemic (H1N1) 2009 by enhanced surveillance afterwards (n = 24).

Case	Age	Sex	Arrival date	Onset date	Days from arrival to onset*	Affected country visited	Body temperature at entry screening (°C)	Contents of health declaration form completed on arrival	Rapid influenza diagnostic test at entry screening	Symptoms on arrival revealed by enhanced surveillance
1	17	F	May 19	May 19	0	USA**	37.8		Negative	Cough, sore throat, nasal obstruction
2	17	F	May 19	May 19	0	USA	39.2		Negative	Sore throat
3	21	F	May 29	May 29	0	USA	38.8		Negative	Cough
4	26	F	May 31	May 31	0	Canada		Cough	N/A	Cough
5	36	M	Jun 2	Jun 2	0	USA	37.2	Cough, nasal discharge, antipyretic	N/A	Cough
6	29	F	Jun 4	Jun 4	0	USA		No symptoms	N/A	Cough
7	32	F	Jun 6	Jun 6	0	USA		Nasal discharge	N/A	Nasal discharge
8	15	M	Jun 6	Jun 6	0	Philippines	40		N/A	
9	48	F	Jun 7	Jun 6	−1	USA		Unknown	N/A	Sore throat
10	33	M	Jun 12	Jun 11	−1	Hawaii	37-	Unknown	N/A	Cough, sore throat
11	15	F	Jun 13	Jun 13	0	Australia	38	Unknown	N/A	
12	27	M	Jun 14	Jun 13	−1	Hawaii		Unknown	N/A	Cough, nasal discharge, nausea
13	41	M	Jun 14	Jun 14	0	Philippines	39	No symptom	N/A	
14	29	F	Jun 14	Jun 14	0	USA	37.3	Unknown	Negative	
15	44	M	Jun 16	Jun 16	0	Philippines		No symptom	N/A	Cough
16	14	M	Jun 16	Jun 12	−4	USA		Unknown	N/A	Cough, sore throat
17	44	M	Jun 16	Jun 16	0	Philippines	38.7	Cough, nasal discharge	N/A	Cough, nasal discharge
18	62	M	Jun 16	Jun 14	−2	Philippines	38	Cough	N/A	Cough
19	31	F	Jun 16	Jun 16	0	USA	38	Unknown	N/A	Sore throat
20	37	F	Jun 16	Jun 16	0	USA	38	Unknown	N/A	
21	38	F	Jun 16	Jun 16	0	USA	>38	Unknown	Negative	
22	3	F	Jun 17	Jun 16	−1	USA	38.5	Unknown	N/A	
23	25	F	Jun 17	Jun 14	−3	Hawaii	38.5	Cough	N/A	Cough
24	28	F	Jun 18	Jun 15	−3	Hawaii		Unknown	N/A	+

Notes: Cases 11 and 13 were detected with an infrared thermoscanner.

Case 8 and 13, Dengue fever was suspected and a test for Dengue was performed.

Case 22 had been negative on a rapid influenza diagnostic test performed in the U.S.

*Days from arrival to onset are expressed as 0 for the day of arrival, as minus number of days for previous days, and as plus number of days for following days.

**USA refers to mainland U.S.

Table 5 shows the number of individuals infected with Pandemic (H1N1) 2009 with or without have a history of international travel, stratified by weeks. Those without such history peaked at 276 during the week of May 15 to 21, decreased the following week, before increasing again thereafter.

**Table 5:** Pandemic (H1N1) 2009-infected individuals with or without history of international travel within 7 days before onset.

	With history of international travel (n = 151)		Without history of international travel (n = 671)	
	n	%	n	%
Period I				
28 April–7 May	3	(75.0)	1	(25.0)
8–14 May	1	(1.7)	59	(98.3)
15–21 May	5	(1.8)	276	(98.2)
Period II				
22–28 May	8	(23.5)	26	(76.5)
29 May–4 June	16	(40.0)	24	(60.0)
5–11 June	38	(20.8)	145	(79.2)
12–18 June	80	(36.4)	140	(63.6)

## Discussion

We have described in detail the border control and community containment measures implemented for identifying individuals with Pandemic (H1N1) 2009 and preventing the spread of infection in the early stages of disease outbreak in Japan, and then assessed the effectiveness of the respective measures based on the data compiled. The data on border control measures used in this study were obtained from the largest international airport in Japan, which accounts for 95% of flights from the affected countries; the data on community containment measures were from reports made to the national authorities by public health centers in accordance with Japan's reporting system for infectious diseases.

We found that among the 471,733 passengers arriving in Japan from affected countries during Periods I and II, 9 infected individuals were picked up by entry screening and 1 was detected during quarantine. As many as 141 individuals who might have been infected during their international travel within 7 days before onset were identified in Japan as the result of the community containment measures. Consequently, 6.6% (10/151) of the individuals infected during international travel were identified by the border control measures. To our knowledge, although there are few reports describing details on the border control measures adopted in other countries, there is an example of strict implementation that was undertaken in China [19]. They strictly enforced body temperature measurement for all passengers arriving from countries at risk. If passengers had fever >37.5°C or one respiratory symptom, they underwent the RT-PCR test. These measures identified 140 of 326 passengers with a history of international travel upon entry in May and June 2009 [19]. Such strict measures are likely not practicable in Japan because of the human resources required to implement such measures. As mentioned earlier, different countries implemented their own border control measures [9], [10]. Because approximately half of the individuals identified in Japan were arriving passengers from the affected countries, it was significant that a large number of these individuals were effectively detected merely by specifying they had been in an affected country. However, 2 individuals among those identified later in Japan to be infected had been missed at entry despite being symptomatic. This was because they had visited the Philippines, not an affected country and therefore Dengue fever was suspected.

Aside from these 2 missed individuals, 22 others were identified after entry into Japan despite being symptomatic at entry screening. Five of them who met the standards of indication for the rapid influenza diagnostic test at entry screening underwent the test and the results were negative. According to a Field Epidemiology Training Program investigation in the National Institute of Infectious Diseases in Japan, the sensitivity of the rapid influenza diagnostic test for Pandemic (H1N1) 2009 was 53.5% [20]. It was reported that the chance of obtaining a negative result was high when the test samples were taken in the early stages after onset [20]. The date of onset these 5 cases were on the day of arrival when indeed these individuals were in the early stages of infection.

Moreover, 3 of these 24 individuals, despite already having symptoms, did not report their symptoms on the health declaration form. One case was detected with an infrared thermoscanner and had an actual axillary body temperature of 39°C. Although there are reports that using infrared thermoscanners might be impractical due to the low detection rate of cases with fever [21], [22], it is a convenient, cost effective, and quick way to screen large groups of people for fever, with the ultimate purpose of identifying fever-related disease of public health significance [23], [24]. In this study, an individual with fever was in fact detected using the infrared thermoscanner although not declaring it on the health declaration form; therefore, it may prove efficient both to check the health declaration forms and use the infrared thermoscanner in order to detect symptomatic individuals. Aho et al. suggested that many of those infected with pandemic (H1N1) 2009 may have been asymptomatic [25]. Therefore, missing asymptomatic cases may be one of causes of infection spreading. Although the infectivity of asymptomatic cases has not been confirmed [26], [27], it may be worthwhile to try to detect cases with fever by using the infrared thermoscanner and the self-reporting health declaration form.

Entry screening in the airplane cabin was adopted as one of the methods for entry screening in Japan, and may have been an effective means of detecting those who had come into close contact with infected individuals (fellow travelers and passengers seated within a radius of 2 m). Identifying fellow travelers is important because, in the present study, infection was detected among some individuals traveling together. Foxwell [28] reported that seats within 2 rows from an infected passenger in an airplane cabin would be in the high-risk range. Furthermore, if an infected individual was onboard for a long flight, the possibility of infection would be small but significant [29]. For possible future outbreaks of pandemic influenza, entry screening in the cabin would enable us to acquire information on passengers located in this high-risk zone. However, it must be noted that of the 206,037 individuals who underwent entry screening in the cabin during Period I in the present study, which required immense human resources to undertake, only 4 were identified as being infected individuals. Trained health staff are finite resources and their deployment to border control and health monitoring efforts reduces the number available for other aspects of pandemic management. This is an important consideration particularly during low prevalence situations. Because entry screening in the cabin for all flights is costly in terms of both money and human resources, in Period II it was limited to only those flights when a symptomatic individual was suspected. According to the Japanese Quarantine Act of 1951 and International Health Regulations of 2005, one of the pilot-in command's duties when a symptomatic individual is found in the cabin before arrival is to report the fact to the quarantine station. If a system in which the pilot could act properly was established, entry screening in the cabin could be carried out for high-risk flights and the human resources required would not be large. Furthermore, if a symptomatic individual in the cabin was reported, the quarantine station could provide instructions to the pilot to order the individual to wear a mask in the cabin and, if possible, sit in a more isolated seat (more than 2 m distant from others) to reduce the risk of spreading by droplet infection [30]–[33].

According to a survey by WHO of 16 countries [9], 56% (9/16) reported that entry screening might have delayed the entry of the Pandemic (H1N1) 2009 virus into their countries, whereas 7 other countries reported that there was no evidence of such effect. Although there are a number of limitations to entry screening methods, the diverse measures of entry screening adopted in Japan might have been effective. Moreover, one individual in the present study who had been diagnosed with Pandemic (H1N1) 2009 virus in the U.S. entered Japan. The need for exit screening, undertaken through international cooperation, should also be considered.

Quarantine aims at the prevention of spreading infection when possibly infected individuals develop symptoms after entering to country. Those in quarantine were offered prophylactic medication with an anti-influenza virus agent. Although the actual number who took the prophylactic medication is unknown, this measure might have reduced the risk of onset of the disease. However, the quarantine system of keeping an individual in a confining facility (e.g., a hotel room or a quarantine unit) has been discussed as a violation of human rights [34]. Reynolds et al. reported that there is a risk that quarantine could cause posttraumatic stress disorder [35].We should consider new methods of quarantine, such as that implemented in Singapore during the SARS outbreak where people were quarantined in their homes [36].

In Japan, entry cards were handed out to all arriving passengers instructing them to consult a public health center if they develop symptoms. Therefore, the public health centers were known as offices in charge of Pandemic (H1N1) 2009 and were relatively easy places for people to access. As more than 90% of the individuals with Pandemic (H1N1) 2009 developed symptoms within 3 days after entry into Japan, it is better to state more clearly on the card that the passengers from at-risk countries should avoid going out at for least 3 days and contact public health centers promptly should symptoms develop.

Health monitoring was expected to contribute to the early detection of cases. At community public health centers, in Period II the staff obtained information from arriving passengers from affected countries by directly calling them. However, although this health monitoring measures was performed across Japan for all 129,546 arriving passengers from the affected countries, only 3 individuals were identified as being infected with Pandemic (H1N1) 2009 during Period I, and there is no evidence indicating the effectiveness of this measures in this Period. In Period II, on the other hand, health monitoring was done mainly for fellow travelers, because they had a high risk of infection, which reduced the number of individuals contacted to 746. Of these, Pandemic (H1N1) 2009 infection was identified in 5 (0.67%), indicating that reducing the number of individuals in Period II was a practical and efficient measure.

Enhanced surveillance was performed to obtain information on the prevalence of infection for proactive epidemiological research, and to help prevent the spread of infection in communities, based on the reports of those individuals identified with the infection. A total of 812 Pandemic (H1N1) 2009 cases were reported through the notification system for infectious diseases and surveillance of infected individuals established in Japan. Furthermore, data on 24 symptomatic individuals not identified upon entry were collected by enhanced surveillance. Conducting such surveillance will enable epidemiological information from Japan to be compared with that from other countries. Such data will also be useful in the assessment of current measures and in establishing new measures for the future.

The first patients of the Pandemic (H1N1) 2009 outbreak in Japan, during the week of May 15 to 21, were high-school students who had no history of international travel [37]. The containment strategy measures of infection imposed in the area included school closure and cancellation of group activities. From May 18 to 21, 180,000 schools were closed [38]. Through careful implementation of these measures, there was a temporary decrease in the number of infected individuals detected in the community [39], [40], a tendency that corresponded with the decrease in new domestic cases across Japan, as detected in this study. These findings suggest that during periods when domestic infection is limited to specific areas, enforcing community containment measures in these affected communities is required. However, the percentage of infected individuals with a history of international travel increased during the period from May 22 to June 4. It is also important to block the entry of infected individuals from overseas to help prevent the infection from spreading.

There are several limitations in this study concerning data collection. In regard to the border control measures, it is not known how many symptomatic individuals were detected on airplanes before arrival. As such, it is impossible to compare the effectiveness of entry screening carried out in the airplane cabin between Periods I and II. As for the community containment measures, there were no data available for the number of attempts made to contact each traveler before they were considered not contactable or the number of travelers who were instructed to go to a health centre and received a rapid diagnostic test. Such quantitative data might be useful for the planning of future public health measures.

The study finding reveal that the border control measures implemented in Japan were effective to a certain degree, because as many as 10 infected individuals were identified using the combined method of a self-reporting health declaration form and an infrared thermoscanner. We believe that each control measure by itself has limitations, and that using several approaches will be more effective. As 18% of the infected individuals identified in Japan had traveled internationally within 7 days before entry, even more active intervention for arriving passengers will be necessary. To implement the current border control measures more effectively, we suggest that it is practical to implement entry screening in the airplane cabin on only those airplanes with symptomatic individuals onboard. For this measure to be implemented, the numbers of passengers to be investigated would need to be investigated to determine what would be feasible practically. In addition, we believe it is important to use the entry cards to explain clearly how arriving passengers should spend the first 3 days after entry, to help prevent the spread of infection. Enhanced surveillance will provide the data important for the improvement of public health measures.

## Materials and Methods

We stated by describing the methods practically adopted in accordance with the contents of a report from the Narita Airport Quarantine Station [41] and notification and implementation guidelines from national authorities, which divided border control measures into entry screening and quarantine, and community containment measures into health monitoring and enhanced surveillance. As the implementation guidelines were revised and measures were changed depending on the spread of the infection in Japan, we described the methods of implementing the measures in detail according to two period: Period I, from April 28 (when WHO declared a pandemic alert phase 4) to May 21; and Period II, from May 22 (when implementation guidelines were revised) to June 18 (when Quarantine officers stopped collecting health declaration forms in accordance with further revision of implementation guidelines).

Regarding entry screening in border control measures, we described the methods used to identify patients in the airplane cabin and the standards for the indication for a diagnostic test. Regarding quarantine, we described the definition of individuals who had come into close contact with infected individuals and the quarantine method adopted.

For border control measures, data were collected from reports prepared by the Narita Airport Quarantine Station at Narita International Airport, and specifically the number of individuals who underwent entry screening, the number of individuals who had the rapid influenza diagnostic test and RT-PCR test to identify Pandemic (H1N1) 2009, the number of positive cases detected, the characteristics of individuals diagnosed with Pandemic (H1N1) 2009 (e.g., date of arrival, date of onset, sex, age, nationality, countries visited, existence of fellow travelers, and body temperature at entry screening). For community containment measures, we described how, and for what individuals, public health centers performed health monitoring. In enhanced surveillance, we described how an infection of an individual was identified and mandatorily reported. Data on community containment measures were obtained from MHLW. The data were originally reported by medical institutions to national authorities via public health centers. These data included the total number of individuals identified as being infected with Pandemic (H1N1) 2009 as revealed by RT-PCR test results, the characteristics of the infected individuals with a history of international travel within 7 days before onset (sex, age, countries visited, and days from arrival to onset), the characteristics of already infected individuals who were not detected by entry screening and whose infection with Pandemic (H1N1) 2009 was identified later in Japan by surveillance (sex, age, date of arrival, date of onset, days from arrival to onset, countries visited, body temperature at entry screening, contents of the health declaration form, with or without a rapid influenza diagnostic test on arrival, and symptoms on arrival).
